# Supplementary material for: Causal Relationship between Aging and Anorexia Nervosa: A White-Matter-Microstructure-Mediated Mendelian Randomization Analysis
Source: Biomedicines. 2024 Aug 16;12(8):1874. doi: 10.3390/biomedicines12081874 (PMC11351342; doi:10.3390/biomedicines12081874)
Supplement: Supplementary file 1 [file biomedicines-12-01874-s001.zip › Supplementary Figure/Figure S7/Single SNP plot of MR analysis for ubm-b-1455 to AN.pdf]

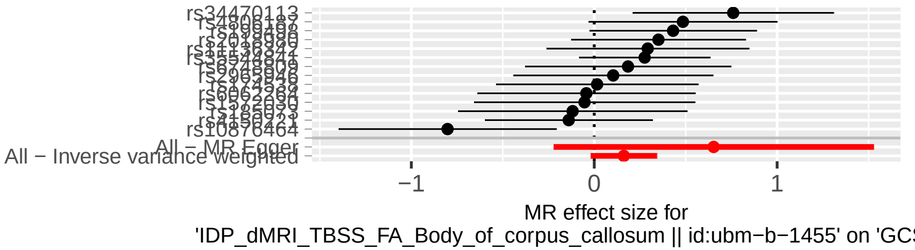

rs34470113
rs10876464
All − MR Egger
All − Inverse variance weighted
−1
0
1
MR effect size for
'IDP_dMRI_TBSS_FA_Body_of_corpus_callosum || id:ubm−b−1455' on 'GCS
